# Efficacy of High-volume Evacuator in Aerosol Reduction: Truth or Myth? A Clinical and Microbiological Study

**DOI:** 10.5681/joddd.2014.032

**Published:** 2014-09-17

**Authors:** Hitesh Desarda, Abhijit Gurav, Chandrakant Dharmadhikari, Abhijeet Shete, Subodh Gaikwad

**Affiliations:** ^1^Post-graduate Student, Department of Periodontology, Tatyasaheb Kore Dental College and Research Centre, New Pargaon, Kolhapur, Maharashtra, India; ^2^Reader, Department of Periodontology, Tatyasaheb Kore Dental College and Research Centre, New Pargaon, Kolhapur, Maharashtra, India; ^3^Professor, Department of Microbiology, Tatyasaheb Kore Dental College and Research Centre, New Pargaon, Kolhapur, Maharashtra, India; ^4^Senior Lecturer, Department of Periodontology, Tatyasaheb Kore Dental College and Research Centre, New Pargaon, Kolhapur, Maharashtra, India

**Keywords:** Aerosols, high-volume evacuator, environmental pollution

## Abstract

***Background and aims.*** Basic periodontal treatment aims at eliminating supra- and sub-gingival plaque and establishing conditions which will allow effective self-performed plaque control. This aim is primarily achieved with sonic and ultrasonic scalers. However, generation of bacterial aerosols during these procedures is of great concern to patients, the dentist and the dental assistant. The aim of this study was to compare the reduction in aerosol with and without high-volume evacuator through a microbiological study.

***Materials and methods.*** For this clinical study a fumigated closed operatory was selected. Maxillary incisors and canines were selected as an area for scaling. Piezoelectric ultrasonic scaling was performed in the absence and in the presence of a high-volume evacuator at 12 and 20 inches from the patient's oral cavity. In both groups scaling was carried out for 10 minutes. Nutrient agar plates were exposed for a total of 20 minutes. After this procedure, nutrient agar plates were incubated in an incubator at 37°C for 24 hours. The next day the nutrient agar plates were examined for colony forming units by a single microbiologist.

***Results.*** The results showed no statistically significant differences in colony forming units (CFU) with and without the use of a high-volume evacuator either at 12 or 20 inches from the patient's oral cavity.

***Conclusion.*** It was concluded that high-volume evacuator, when used as a separate unit without any modification, is not effective in reducing aerosol counts and environmental contamination.

## Introduction

Basic periodontal treatment aims at eliminating supra- and subgingival plaque and this aim is primarily achieved with hand-held instruments and sonic and ultrasonic scalers.^1^ However, generation of bacterial aerosols is a concern for patients, staff and practitioners.^2,3^

Different techniques are documented in the literature to minimize/eliminate the hazard produced due to the aerosols, like the use of personal barriers such as masks, gloves and eye protection.^4,5^ Airborne contamination arising from the operative field during the periodontal procedures can be efficiently reduced by the use of a high efficiency particulate air (HEPA) filter, ultraviolet (UV) chambers in the ventilation system,^6^ and a high-volume evacuator (HVE).^7,8^ But very limited data is available regarding the efficacy of high-volume evacuator and most reports describe the modification of it, which is not suitable during routine dental practice.

Therefore the aim of the present study was to evaluate the efficacy of high-volume evacuator without any modification to reduce the aerosol contamination of the operating field during scaling of chronic generalized periodontitis patients.

## Materials and Methods

In this clinical study, 80 subjects with the diagnosis of chronic generalized periodontitis were selected from the Outpatient Department (OPD) of the Department of Periodontology. The study was reviewed and approved by the Institutional Ethics Committee.

### Inclusion Criteria

- Age range: 30−60 years
- Patients with chronic generalized periodontitis were selected based on International Workshop for Classification of Periodontal Diseases, 1999 (AAP 1999)

### Exclusion Criteria

- Patients with severely debilitating systemic diseases
- Patients on any antibiotics since the previous six-month period
- Patients with pacemakers
- Patients with a history of respiratory diseases
- A history of previous periodontal treatment since the previous 1-year period

### Sample Selection

Eighty subjects fulfilling the inclusion criteria were selected for the study. Informed written consent was taken from the enrolled patients. Periodontal status was evaluated by a UNC-15 probe. The following parameters were recorded in a predesigned form.

- Oral Hygiene Index-Simplified (Greene and Vermillion, 1964)
- Plaque index (PI) (Silness and Loe,1964)
- Probing depth
- Recession
- Clinical attachment level

### Procedure

For this clinical study a closed operatory was selected, which was fumigated overnight prior to starting the procedure. Fumigation was performed by using a fumigator machine (OT Formalin Aerosol^TM^). The setup was kept constant throughout the course of the study.

Nutrient agar plates were prepared for microbiological analysis with all the precautions to avoid any possible contamination. All the agar plates were kept in a plastic container which was thoroughly cleaned with a surface disinfectant (TORCILOL RAPID^TM^) and preserved in a refrigerator in a separate compartment. Another similar plastic container was used to transport the agar plates from the incubator to the operatory and vice versa. Prior to starting the procedure, five agar plates were removed from the refrigerator and kept open in an incubator for 20 minutes at 37°C to allow the vaporization of the liquid layer which was formed due to freezing. Out of these five plates one plate was used as a control to evaluate any contamination of agar plates during preparation. The remaining four plates were used during the procedure.

**Figure 1.**
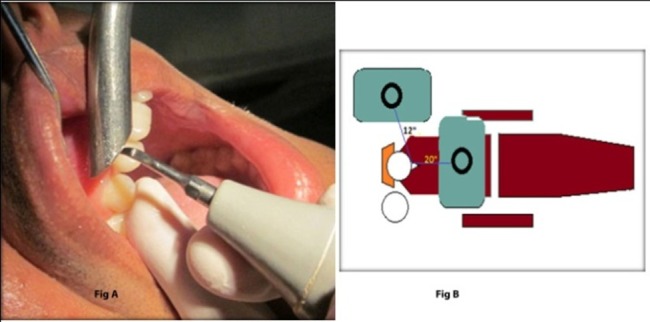
(A) Position of scaler tip and high-volume evacuator tip; (B) Diagrammatic representation.

Maxillary incisors and canines were selected as an area for scaling. A piezoelectric scaler (BONART^TM^) was used for the study. Scaling was performed in the absence of a high-volume evacuator (control group, group X) and in the presence of the high-volume evacuator (study group, group Y) in the above-mentioned area. Coin toss was used to determine which procedure was to be performed first (i.e. with high-volume evacuator or without high-volume evacuator). Power and water flow settings of the scaler was kept the same throughout the procedure for both groups. The high-volume evacuator tip used in this study was stainless steel with a diameter of 12 mm (Figure 1A). A disposable saliva ejector was used for saliva ejection. No pre-procedural rinse was advised to any of the patients.

A total of four nutrient agar plates were used for microbial sampling, out of which two were used for the control and two for the study groups. From these nutrient agar plates, one plate was exposed at 20 inches from the patient’s oral cavity in front of the patient and another plate was exposed at 12 inches from the patient’s oral cavity at assistant side in the control group. Similarly, the other 2 plates were placed for the study group. The operator side was not assessed as the operator had to move continuously throughout the procedure (Figure 1B).

Two holding stands were prepared for the placement and exposure of agar plates. In both groups (control and test groups) ultrasonic scaling was carried out for 10 minutes. Nutrient agar plates were exposed for a total of 20 minutes. The investigator, assistant and the subject were still in their position for 10 minutes after ultrasonic scaling to prevent any air turbulence that could cause dispersion of aerosol particles. After this procedure, nutrient agar plates were incubated in an incubator at 37°C for 24 hours. The next day, the nutrient agar plates were examined for colony forming units by a single microbiologist who was unaware of the procedure performed. The readings were then recorded on the designed form.

### Statistical Analysis

Statistical software programs, namely SAS 9.2, SPSS 15.0, Stata 10.1, MedCalc 9.0.1, Systat 12.0 and R Environment 2.11.1, were used for the analysis of data and Microsoft Word and Excel were used to generate graphs and tables.

## Results

Statistical analysis clearly demonstrated that there was no statistically significant difference in the number of CFUs (P = 0.248) between group X (11.08 ± 2.25) and group Y (12.14 ± 1.93).

In addition, there was no statistically significant difference in the number of CFUs (P=0.154) at 12 and 20 inches from the patient’s oral cavity when high-volume evacuator was not used. Similarly no statistically significant difference was noted in the number of CFUs (P=0.155) with the use of a high-volume evacuator.

**Figure 2.**
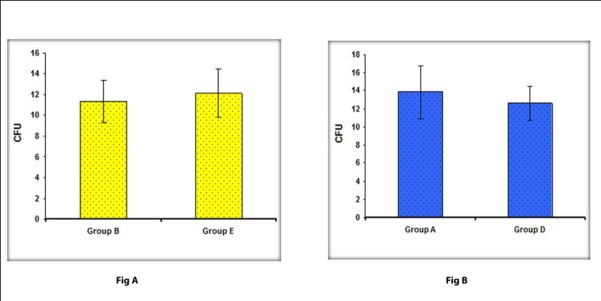
(A) Comparison of CFU between group A and group D; (B) Comparison of CFU between group B and group E.

Differences in the reduction in colony counts at 12 (Figure 2B) and 20 inches (Figure 2A) with and without a high-volume evacuator were evaluated with Student’s t-test and significance was assessed at 5% level of significance. Statistical analysis showed no statistically significant differences in the number of CFUs either at 12 inches (P=0.49) or 20 inches (P=0.617).

## Discussion

The main sources of environmental contamination during dental procedures are aerosols and splatter.^9,10^ The terms “aerosol” and “splatter” in the dental environment were used by Micik and colleagues^7^ in their pioneering work on aerobiology. They defined aerosols as particles less than 50 micrometers (μm) in diameter. Particles of this size are small enough to stay airborne for an extended period before they settle on environmental surfaces or enter the respiratory tract. Splatter was defined as airborne particles larger than 50 μm in diameter.^7^ They stated that splatter particles behaved in a ballistic manner. This means that these particles or droplets are ejected forcibly from the operating site and arch in a trajectory similar to that of a bullet until they contact a surface or fall to the floor.^7^ Aerosols, splatter and droplet nuclei have been implicated in the transmission of diseases like tuberculosis (TB),^11^ diseases of viral origin,^12^ pneumonitis^12^ and influenza.^13^

It is interesting to note that most of the studies supporting the use of high-volume suction in aerosol reduction reported the use of modified devices. To our knowledge this is the first in vivo study evaluating the effectiveness of a high-volume evacuator without any modification to simulate the clinical scenario.

The results of this study showed no statistically significant differences in colony forming units (CFU) with and without the use of a high-volume evacuator either at 12 or 20 inches from patient’s oral cavity.

These results were in contrast to King et al,^13^ who found that ultrasonic scaler without the aerosol reduction device (ultrasonic scaler alone) had a significantly greater quantity of mean colony forming units (CFUs) 6 inches from the subject's oral cavity than the ultrasonic scaler with the aerosol reduction device (i.e. combination of HVE and ultrasonic scaler). The differences in the results might be attributed to the fact that King et al used modified device which may have blocked the splatter from reaching the agar plates. Muzzin et al^11^ in their in vivo study used a modified device consisting of a high-volume evacuator and air polisher as a single unit and reported contrasting results showing a 86% reduction in CFU.

In another study, Yamada et al^14^ evaluated the effect of high-volume evacuator in reducing aerosol blood mist. The study showed that the use of double extraoral high-volume evacuator system was more beneficial in reducing aerosol blood mist as compared to single extraoral evacuator system. The results of the present study clearly showed that high-volume evacuator is not very effective in reducing the aerosol count. Splatter might have played a key role in environmental contamination, but it was not considered in previous studies. Splatter causes environmental contamination because of its high kinetic energy; it travels against the air current and is not eliminated by the use of a high-volume evacuator. Thus, other methods should be considered to reduce aerosol contamination^5,7^ in addition to universal barrier precautions.

## Conclusion

Within the limitations of the present study, the results showed no difference in reduction of aerosols with or without the use of a high-volume evacuator when analyzed microbiologically. Thus it was concluded that high-volume evacuator when used as a separate unit without any modification is not effective in reducing aerosol count and environmental contamination.

We may take the liberty at this conjuncture to point at the need to develop a simple, effective, cheap and reliable alternative for reduction of environmental contamination by both aerosols and splatter, which would be beneficial and feasible to use in a day-to-day clinical setting.
